# Photochemical Energy Conversion with Artificial Molecular Machines

**DOI:** 10.1021/acs.energyfuels.1c02921

**Published:** 2021-10-01

**Authors:** Leonardo Andreoni, Massimo Baroncini, Jessica Groppi, Serena Silvi, Chiara Taticchi, Alberto Credi

**Affiliations:** † CLAN-Center for Light Activated Nanostructures, Istituto ISOF-CNR, Via Gobetti 101, 40129 Bologna, Italy; ‡ Dipartimento di Chimica “G. Ciamician”, Università di Bologna, Via Selmi 2, 40126 Bologna, Italy; § Dipartimento di Scienze e Tecnologie Agro-alimentari, Università di Bologna, Viale Fanin 50, 40127 Bologna, Italy; ∥ Dipartimento di Chimica Industriale “Toso Montanari”, Università di Bologna, Viale del Risorgimento 4, 40136 Bologna, Italy

## Abstract

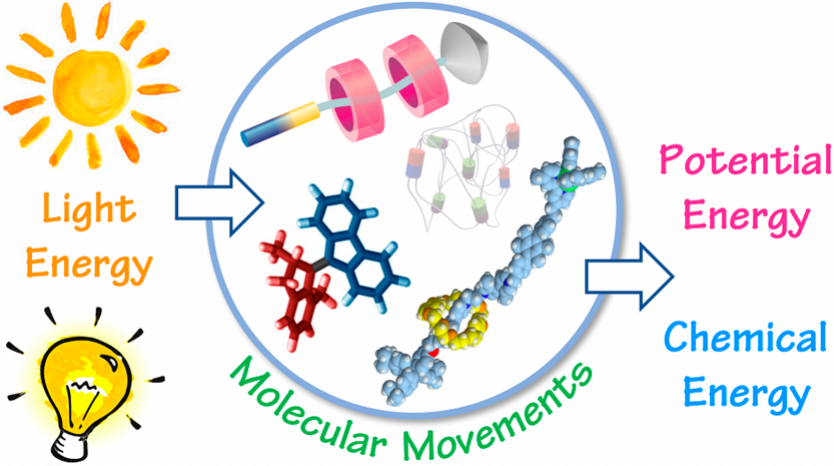

The exploitation of sunlight as a clean, renewable, and distributed energy source is key to facing the energetic demand of modern society in a sustainable and affordable fashion. In the past few decades, chemists have learned to make molecular machines, that is, synthetic chemical systems in which energy inputs cause controlled movements of molecular components that could be used to perform a task. A variety of artificial molecular machines operated by light have been constructed by implementing photochemical processes within appropriately designed (supra)molecular assemblies. These studies could open up new routes for the realization of nanostructured devices and materials capable to harness, convert, and store light energy.

## 1 Introduction

In our macroscopic world, we make large use of mechanical machines. These devices can do mechanical work, by means of controlled movements performed upon supplying energy from a source, which is typically a fuel (i.e., chemical) or electricity. The work done by machines is used to accomplish tasks that frequently involve conversion into another form of energy. A typical example is an electrically powered pump employed to transfer a mass of water uphill; the electrical energy input is converted into mechanical work (movement), which in its turn is transformed in gravitational potential energy.

Macroscopic machines are assemblies of different parts that work together to execute a function. In the context of research on supramolecular chemistry, which deals with “the organized entities of higher complexity that result from the association of two or more chemical species held together by intermolecular forces”,^1^ following early conceptual discussions of physicist Richard Feynman^2^ and engineer Eric Drexler,^3^ it was proposed that the notion of device and machine can be transferred to the molecular scale.^1,4,5^ In brief, a molecular device can be considered as an assembly of a defined number of molecular components designed to execute a function upon appropriate external stimulation. A molecular machine is a particular class of molecular device where the function is achieved by means of the mechanical movement of its molecular components.^1,5,6^

Supramolecular chemistry is an area of chemistry closely related to biology that has expanded intensely over the past 40 years.^1,7,8^ Owing to the progress of chemical synthesis, supramolecular systems composed of a significant number of molecular components capable of self-assembling in a predetermined fashion can nowadays be designed and prepared. Assemblies in which distinct molecular components are held together by covalent or coordination bonds, as in grids, racks, arrays, or dendrimers, or even mechanically interlocked with each other, as in rotaxanes and catenanes, can also be constructed.^7^ Although such systems would not fall into the original definition of supramolecular species given in the previous paragraph, it was pointed out that they can exhibit a supramolecular behavior with regard to their physicochemical properties and the related emerging functions.^6^ The common element of all these species is their multicomponent nature, that is, the fact that they consist of molecular units linked by means of interactions that span from weak electrostatic forces to strong covalent bonds.

The impetus to build molecular devices and machines comes in a large part from the extraordinary advances in molecular biology, thanks to which we are beginning to comprehend the secrets of the natural nanomachines that underpin life.^9,10^ The supramolecular architectures of the biological world are certainly the main and proven examples of the feasibility and usefulness of nanotechnology.^10,11^ Their efficiency constitutes a strong motivation for engaging in the realization of artificial molecular devices. The bottom-up construction of sophisticated devices and machines such as those found in nature, however, is currently a prohibitive task. Therefore, chemists have sought (i) to build much simpler systems, without mimicking the complexity of biological structures,^12^ (ii) to understand the principles and processes underlying their functioning,^13^ and (iii) to address the challenges inherent in the interfacing of these systems with the macroscopic world, particularly concerning energy supply^14^ and information exchange.^15^

**Figure 1:**
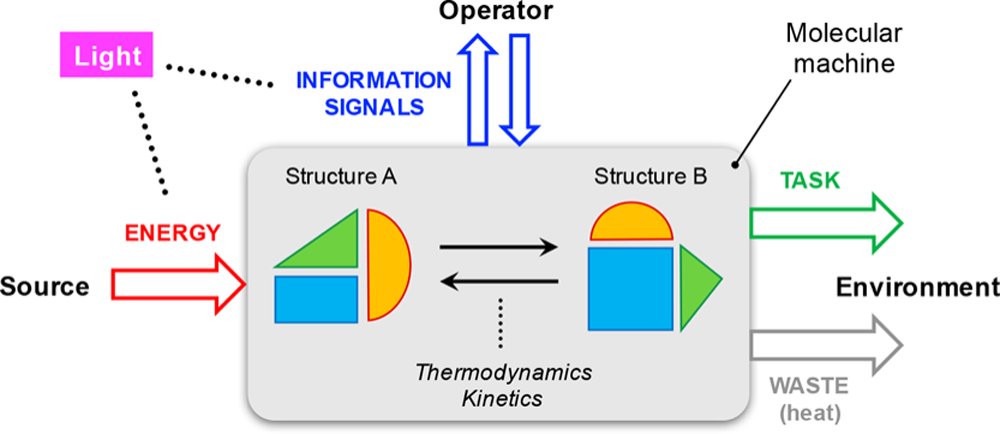
Schematic representation of light-fueled molecular machines. Photons can be used both to deliver energy to the system and to gain information about its state. The operation mechanism involves transitions between at least two structurally different states, with light potentially affecting both the thermodynamic and kinetic aspects of such transformations.

Molecular devices and machines, similarly to their macroscopic counterparts, need to be fed with energy and to exchange signals with their environment (e.g., with an external operator) (Figure 1). Such a dual requirement could indeed be satisfied by light because photons are capable of triggering endergonic transformations in the system, and at the same time, their interaction with the latter can provide information on its state (e.g., with spectroscopic methods).^16,17^ This is also true in nature, where living systems utilize sunlight photons as both energy quanta in photosynthesis and as information elements in vision-related processes.^18^

This short review is aimed at highlighting the potential of artificial molecular machines to harvest, convert, and utilize light energy to perform tasks. We first recall a few basic concepts of molecular machines and the implications of their light-driven operation. Then, we describe a number of selected examples that illustrate the strategies explored and the prominent results obtained in the field. Limitations, open problems, and future directions are discussed in the final part.

## 2 Artificial Molecular Machines

A molecular machine can be defined as an assembly of a discrete number of molecular components that exhibit controlled mechanical movements in response to an external stimulus. Molecular switches and motors are classes of molecular machines, which, as pointed out in the Introduction, belong to the broader category of molecular devices.

During the past four decades, the application of an engineering mentality to research in synthetic, physical, and analytical chemistry has led to the development of a large variety of artificial molecular machines and motors, utilizing a wide range of molecular and supramolecular species, both manmade and of biological origin. The key concepts, most significant achievements, and perspectives of such systems have been discussed in a few monographs^19−21^ and edited books^22−24^ and in several excellent reviews.^25−31^ The high scientific value of this research, and its potential to enable revolutionary applications in several areas of technology and medicine,^32−38^ was recognized in 2016 by the award of the Nobel in Prize in Chemistry to Jean-Pierre Sauvage, Fraser Stoddart, and Ben Feringa “for the design and synthesis of molecular machines”.^39^

### 2.1 Basic Concepts

The design and construction of artificial molecular machines requires knowledge of the chemical–physical principles that govern the functioning of the natural versions.^40^ The observation of biological nanodevices, in fact, reveals that molecular machines should not be considered simply as shrunken versions of their macroscopic counterparts.^8,9^ While the latter are made of hard and rigid materials and can rely on temperature differences to operate (as in internal combustion engines), molecular machines are made up of soft components and must operate at a constant temperature (determined by the environment), because, as noted by Feynman in his historic speech on what later became known as molecular nanotechnology,^2^ heat flows very quickly at the nanoscale. Indeed, most biomolecular motors in our body—for example, myosin and kinesin—are powered by an exergonic chemical reaction, ATP hydrolysis, that happens at constant temperature.

Due to the tiny mass of molecules, gravity and inertial effects are negligible on the molecular scale, where viscous forces arising from intermolecular interactions (including those with solvent molecules) dominate. A distinctive feature of the molecular scale with regard to motion is the fact that objects of nanometer size are subject to the incessant and random agitation caused by thermal energy (Brownian motion). The second law of thermodynamics states that such a movement—which is unavoidable, unless at absolute zero—cannot be harnessed to produce useful work. Under normal conditions, Brownian motion has a disruptive effect on the movement of molecular species; it can be estimated that the thermal noise power to which a molecule is subjected at ambient temperature is 10^–8^ W, that is, at least 8 orders of magnitude greater than the power supplied by the chemical reaction that powers a biomolecular motor.^41^ In other words, the problem of making molecules move in a controlled manner resembles that of riding a bike during an earthquake; since the latter cannot be stopped, the sole viable solution is to exploit the shakes to move forward. This is in fact what biological molecular motors do; they exploit an external energy source to bias random Brownian motion, thus rendering the movement in a given direction more likely than that in others.

**Figure 2:**
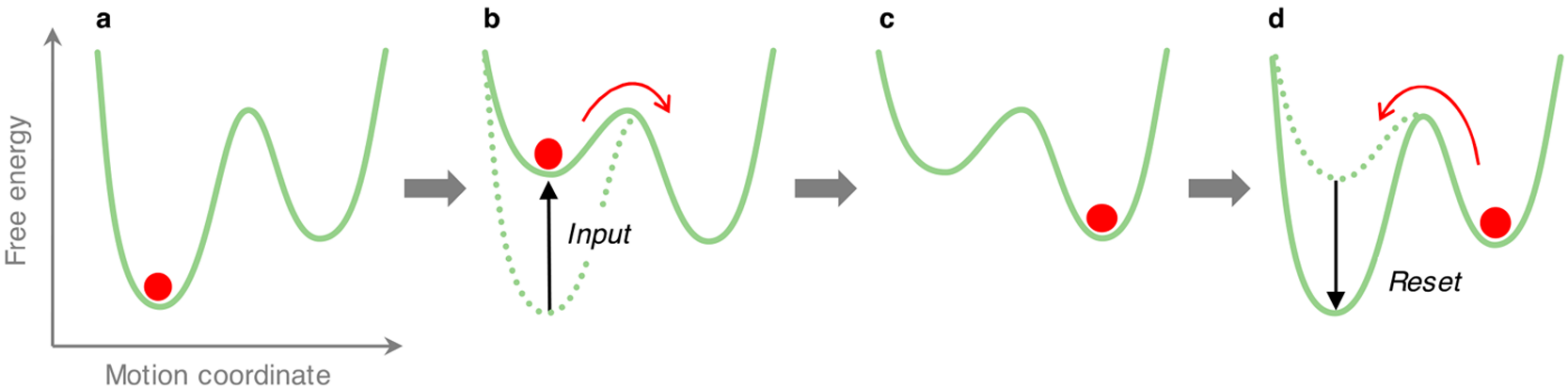
Schematic representation of the directional motion of a molecular-scale object (red circle) caused by an energy input. The object initially resides in the absolute free energy minimum (a), an external stimulus destabilizes the starting state with respect to a nearby local minimum (b), and thermal agitation moves the object directionally until a new equilibrium is reached (c). Reset of the energy profile causes the return of the Brownian object back to the initial position (d).

Figure 2 provides a simple illustration of this principle for a one-dimensional system. The object is initially positioned in the global free energy minimum (a); in order to move it to the next well, an external energy input is necessary. Such an input, however, does not need to be so large to overcome the barrier; it should only make the starting state less stable with respect to the next relative minimum (b). In such a case, the system goes out of equilibrium, and Brownian motion will push the object over the (decreased) barrier to reach the new global minimum, thus re-establishing equilibrium (c). Therefore, the motion is actually powered by thermal agitation, with the external input providing directionality. When the perturbation ends, or an opposite input is activated, the free energy profile is reset, and the object returns on the original position, again taking advantage of Brownian motion (d). More details on the mechanisms by which energy stimuli can be exploited to rectify random thermal motion—for example, how the energy released in ATP hydrolysis is converted into mechanical work in biomolecular motors—can be found elsewhere.^8,9,11,27−29,38,40^

From this discussion, it is clear that accomplishing controlled and directional movements at the molecular scale is a challenging objective, and a careful design of the systems and their stimuli-induced transformations—keeping in mind the phenomena mentioned above—is required.

### 2.2 Light as an Energy Supply

Although sunlight is the primary energy source for all living systems, the direct conversion of light energy into motion is quite rare in biological systems; an example is bacteriorhodopsin, wherein photoisomerization triggers conformational changes that ultimately make protons pass across a membrane.^42^ Sunlight is typically converted into high energy chemicals by photosynthetic processes;^43,44^ these species are subsequently transformed into appropriate “fuels” (e.g., ATP) which are utilized in the end to power all biological functions, including those involving movements.

Chemical fuels are particularly suited to meet the daily energy needs of living organisms because they can be conveniently stored, transported, and processed. A device that exploits chemical energy, however, will require fresh reactants to be available at every step of its functioning cycle, and waste products will be concomitantly generated. The progressive accumulation of this “waste” will compromise the functioning of the device, unless it is removed, as happens both in our cells and in internal combustion engines. It is clear that the need to dispose of waste products poses considerable problems in the design and construction of artificial molecular machines driven by chemical inputs. On the other hand, it is well known that light energy inputs can cause reversible and “clean” endergonic reactions.

Feeding artificial molecular machines with light energy exhibits further advantages compared to chemical or electrical stimulation.^17,45^ First of all, if the absorption spectrum of the species of interest is known, the amount of energy supplied can be carefully controlled by means of the wavelength and intensity of the exciting light. The energy of photons can be transferred to molecules without “wiring” them to the source; the only requirement is that the matrix is transparent at the excitation wavelength. Other properties of light, such as polarization, can also be harnessed. Modern techniques and light sources provide the opportunity to limit excitation to very small spaces and extremely short times, whereas the irradiation of large areas and volumes enables the simultaneous stimulation of a huge number of individual nanomachines.

Because of the extremely small size of molecules, the observation of motion is not a trivial issue in research on molecular machines. In general, the movement of the component parts should cause readable changes in some chemical or physical properties of the system. Photochemical interactions are also useful in this regard, because optical spectroscopic methods (for example, luminescence spectroscopy) can provide information on the state of the machine. Hence, similarly to natural systems, it is also true for artificial molecular machines that photons can play the dual role of *writing* (i.e., causing a change in the system) and *reading* (i.e., reporting the state of the system).^14,15^

The fact that photoinduced processes can directly bring about motion and generation of force, thus deterministically relating the energy input with the “power stroke” of the device, is a remarkable feature of light-driven molecular machines.^46^ A further element of interest about optical stimulation is that the use of reversible photochemical processes in appropriately designed molecules can enable the development of motors that function autonomously, which means that they can repeat their functioning cycle under constant experimental conditions as long as light is supplied. This result is achieved by devising a mechanism based on a cyclic pathway that leads the system through transient electronic and structural (mechanical) states, in which at least one of the steps is photoinduced. For instance, the operation of the machine could be based on a photoinduced sequence of processes that involve electronically excited states, where the final deactivation of the system to the ground state provides an automatic reset that closes the cycle. Alternatively, the mechanical motion could be related to the light-triggered switching between stable and metastable states, as it happens in photochromic systems. In both cases, once the system has performed one complete cycle, the successive absorption of another photon triggers a new cycle, and the process can be repeated indefinitely at a frequency that depends on the time scale of the transformations comprised in the operating cycle (unless irradiation is performed at such a low intensity that the flux of incoming photons determines the reaction rate). These paradigms will be conveniently illustrated with the case studies discussed in the next section.

In order to highlight the role of molecular machines for processing light energy, and also for space reasons, in this review we deal only with cases in which the photochemical energy conversion is intrinsic to the molecular machine. Hence, systems and materials wherein the photomechanical actuation at the basis of energy conversion and storage stems from bulk effects (e.g., anisotropy of the matrix) are not discussed; interested readers can refer to specific recent articles^47−50^ and reviews.^51−54^

## 3 Case Studies

### 3.1 Molecular Shuttles Driven by Light

Molecular shuttles are certainly the most popular realization of the molecular machine concept with artificial chemical species.^55^ These machines are based on rotaxanes, i.e., mechanically interlocked molecules (MIMs)^20^ minimally composed of a macrocyclic ring encircling a molecular axle endowed with terminal bulky groups to prevent dethreading. MIMs are very appealing platforms to develop molecular machinery for the following reasons: (i) the molecular components, due to the lack of strong chemical bonds between them, can easily undergo rearrangements; (ii) the mechanical bond confines the intercomponent movements within a defined range and ensures the overall stability of the system; (iii) the population of a specific mutual arrangement of the components is determined by the strength of noncovalent interactions between them; (iv) such interactions can be modulated by external stimulation.

In a molecular shuttle, the ring component moves in a linear fashion along the molecular axle, in a way that reminds the operation of an abacus. If there is no obstacle on the axle, Brownian motion will cause the ring to move back and forth in a random fashion. The simplest design of a controllable molecular shuttle—i.e., a system wherein the position of the ring along the axle can be set by external stimulation—implies the presence of two different recognition sites (stations) on the axle. The ring initially encircles the most efficient station until a chemical reaction, properly activated, changes the relative affinity between the ring and the stations, thereby causing the thermally driven movement of the ring to the other station according to the mechanism discussed in Figure 2.^19−34^

**Figure 3:**
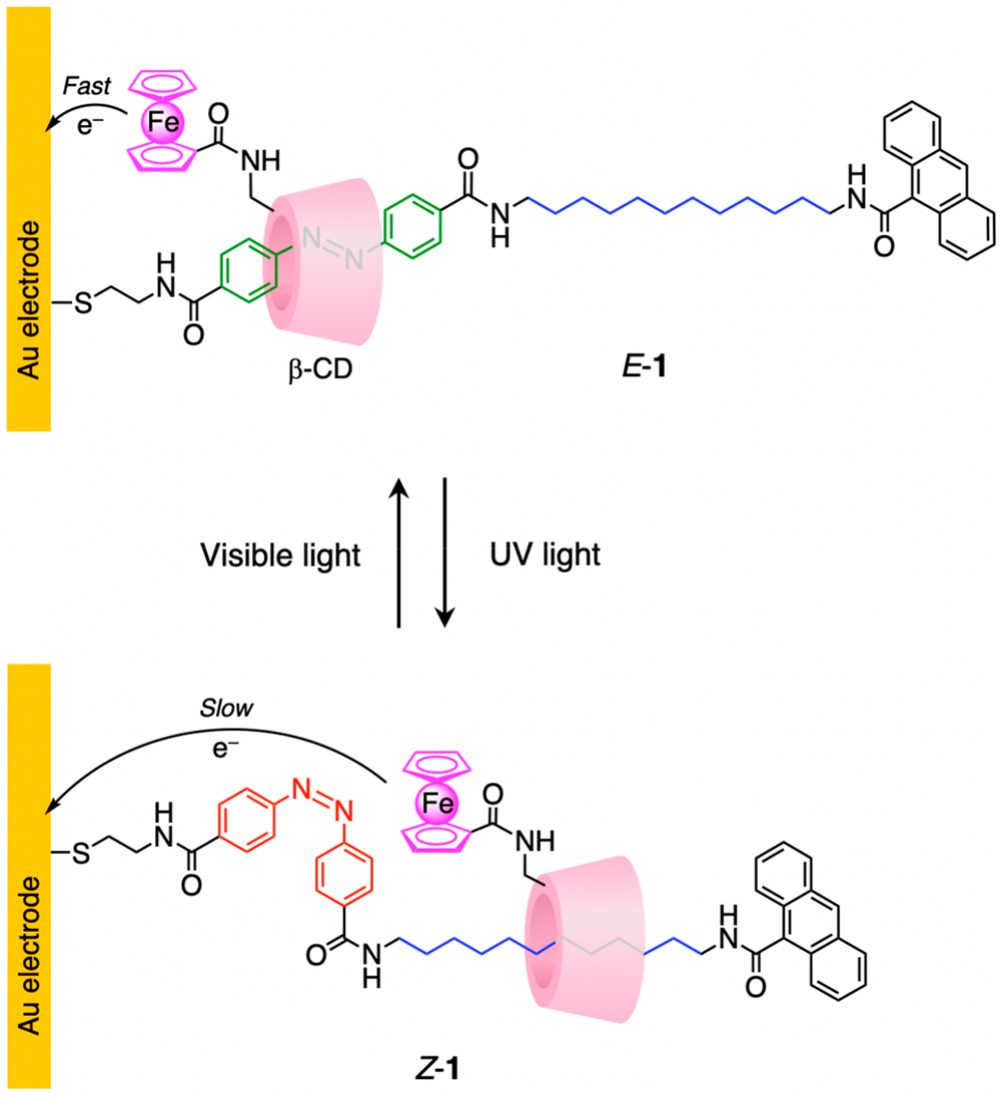
Rotaxane **1** is a photochemically driven molecular shuttle in which an optical (ultraviolet light) stimulus is transduced into an electrical signal by means of mechanical movements.^58^

Photoinduced reactions such as isomerization^56^ and electron transfer^57^ have been widely employed to modulate the interaction of the ring with the stations with the purpose of causing mechanical movements. A relatively dated but highly instructional example based on the reversible *E-Z* photoisomerization of azobenzene is rotaxane **1** (Figure 3), which was deposited as a self-assembled monolayer on a gold electrode.^58^ The ring is interlocked because the threaded axle is bound to the surface on one end and bears an anthracene stopper on the other end. The azobenzene unit, initially in the *E* configuration, is complexed by the ferrocene-functionalized β-cyclodextrin (β-CD). Upon isomerization to the *Z* form, induced by irradiation with ultraviolet (UV) light, the encapsulation becomes sterically impossible, and the β-CD macrocycle has to move toward the alkyl spacer. Back *Z* → *E* isomerization, caused by exposure to visible light, is followed by the return of the ring at the original location. As the current intensity associated with oxidation of the appended ferrocene unit depends on its distance from the electrode, the position of the β-CD ring along the axle could be determined electrochemically. Interestingly, this nanomechanical device exploits a shuttling motion to transduce optical information (light input) into an electrical signal (current output).

In a related investigation,^59^ the local change in wettability of a surface, caused by UV irradiation and mediated by ring shuttling in surface-bound rotaxanes, was exploited to move a small liquid drop up an incline, thereby using light energy to do work against gravity at the macroscopic scale. In brief, the axle of the rotaxane contains a primary fumaramide and a secondary tetrafluorosuccinamide stations; the former one can be photoisomerized to the maleamide form, which has a very low affinity for the employed tetralactam macrocycle. Hence, in the dark, the ring encircles the fumaramide site, while it is shifted to the fluorinated succinamide site upon UV irradiation. Interestingly, the photoinduced ring shuttling in this system leads to exposure or concealment of the highly hydrophobic fluoroalkane portion of the axle, thereby changing the surface tension. In the key experiment, a portion of surface next to a 1.25 μL (4.16 μg) diiodomethane droplet was exposed to light; the liquid was attracted toward the irradiated area and thus moved across the surface. The droplet was transported for 1.38 mm up a 12° incline, resulting in 1.2 × 10^–8^ J of work done against gravity. Considering that the droplet subtends ca. 2 × 10^12^ rotaxane molecules and the photoswitching efficiency is 40%, each molecular shuttle contributes with 1.5 × 10^–20^ J, i.e., 9 kJ mol^–1^. It should be noted, however, that the droplet does not move as a direct consequence of amplified molecular-scale movements and that similar results were obtained with simple photochromic compounds deposited on surfaces.^60^

**Figure 4:**
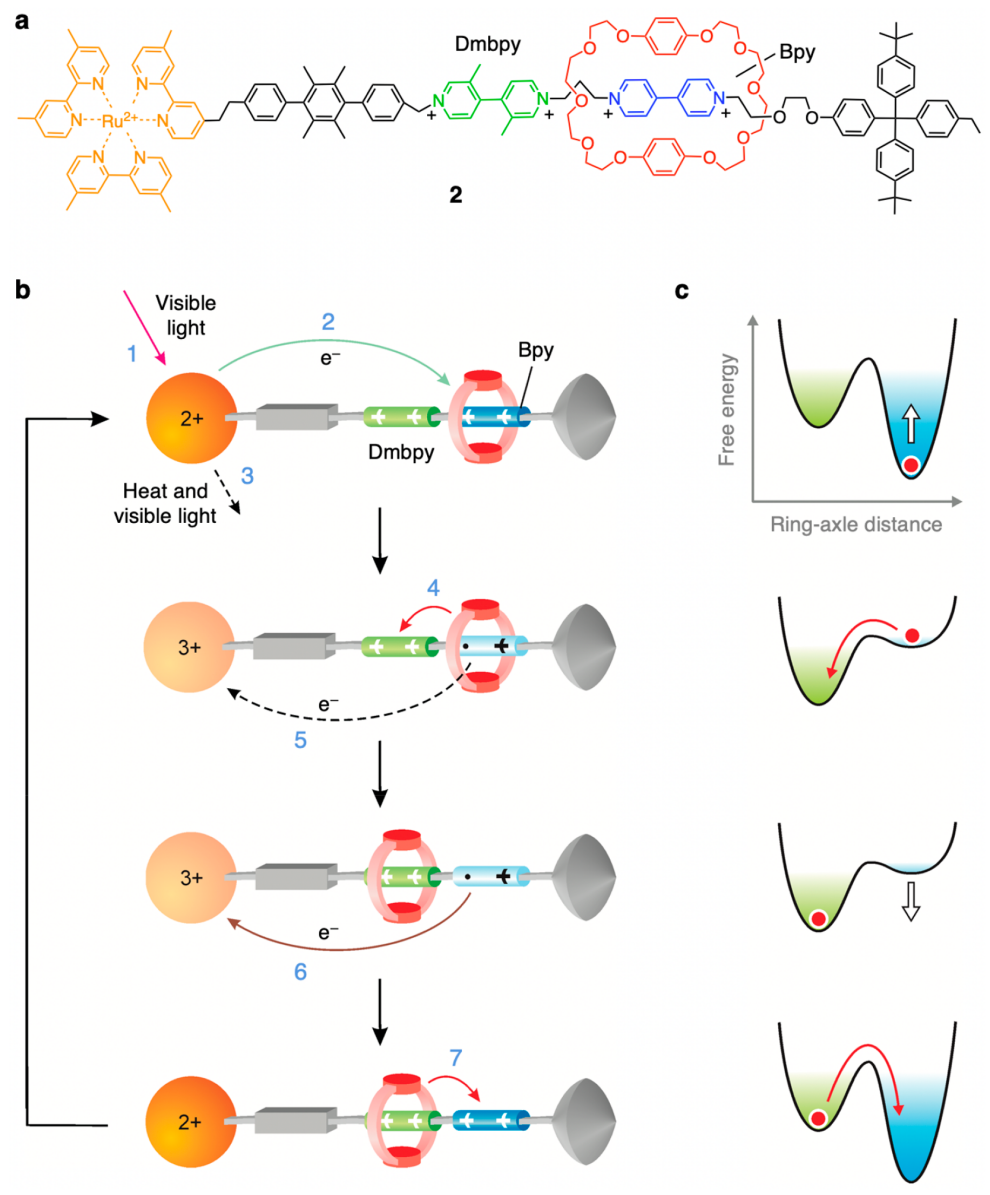
Structure formula of rotaxane **2** (a) and schematic representation of its reversible and autonomous ring shuttling powered by light (b).^61^ The operation is based solely on intramolecular processes, and no waste is produced. Dashed lines represent undesired competing processes. In analogy with the curves shown in Figure 2 and related discussion, the simplified free energy profiles corresponding to each structure in (b) are shown in (c).

The ability of the rotaxanes just described to autonomously repeat their shuttling cycle under constant irradiation, however, was not demonstrated. A molecular shuttle designed to exhibit reversible and autonomous ring shuttling powered solely by visible light is compound **2** (Figure 4).^61^ In this case, the macrocycle is a π-electron-rich crown ether, whereas the axle comprises several covalently linked units, namely, a Ru(II) polypyridine complex, a *p*-terphenyl-type rigid spacer, π-electron-poor 4,4′-bipyridinium (Bpy) and 3,3′-dimethyl-4,4′-bipyridinium (Dmbpy) units, and a tetraarylmethane group as the terminal stopper (Figure 4a). The ruthenium-based moiety plays the dual role of a photosensitizer and a stopper, whereas the mechanical switch consists of the two electron-poor recognition sites and the electron-rich macrocycle. In the stable state, the ring encircles the Bpy site because it is a more efficient electron acceptor than the Dmbpy one.

As illustrated in Figure 4b and c, the light-fueled shuttling of the macrocycle between the Bpy and Dmbpy stations is based on a four-step sequence of electron transfer and molecular rearrangement processes. In brief, selective excitation of the Ru-based unit of **2** with visible light (step 1) affords a triplet excited state which is sufficiently reductant and long-lived to transfer an electron to the Bpy site (step 2), in competition with the intrinsic decay of the excited state (step 3). The reduced Bpy station becomes deactivated, and as a consequence, Brownian motion moves the macrocycle by 1.3 nm to reach the Dmbpy unit (step 4). This process competes with the back-electron transfer from the reduced Bpy unit, still encircled by the ring, to the oxidized photosensitizer (step 5). Once the macrocycle has moved away from its initial location, back-electron transfer from the reduced and uncomplexed Bpy to the oxidized Ru-based unit (step 6) regenerates the primary station by restoring its electron acceptor properties. As a consequence of such an “electronic reset”, the ring moves from Dmbpy back to Bpy by Brownian motion (step 7). The cycle is thus completed, and the rotaxane is ready to process another photon.

**Figure 5:**
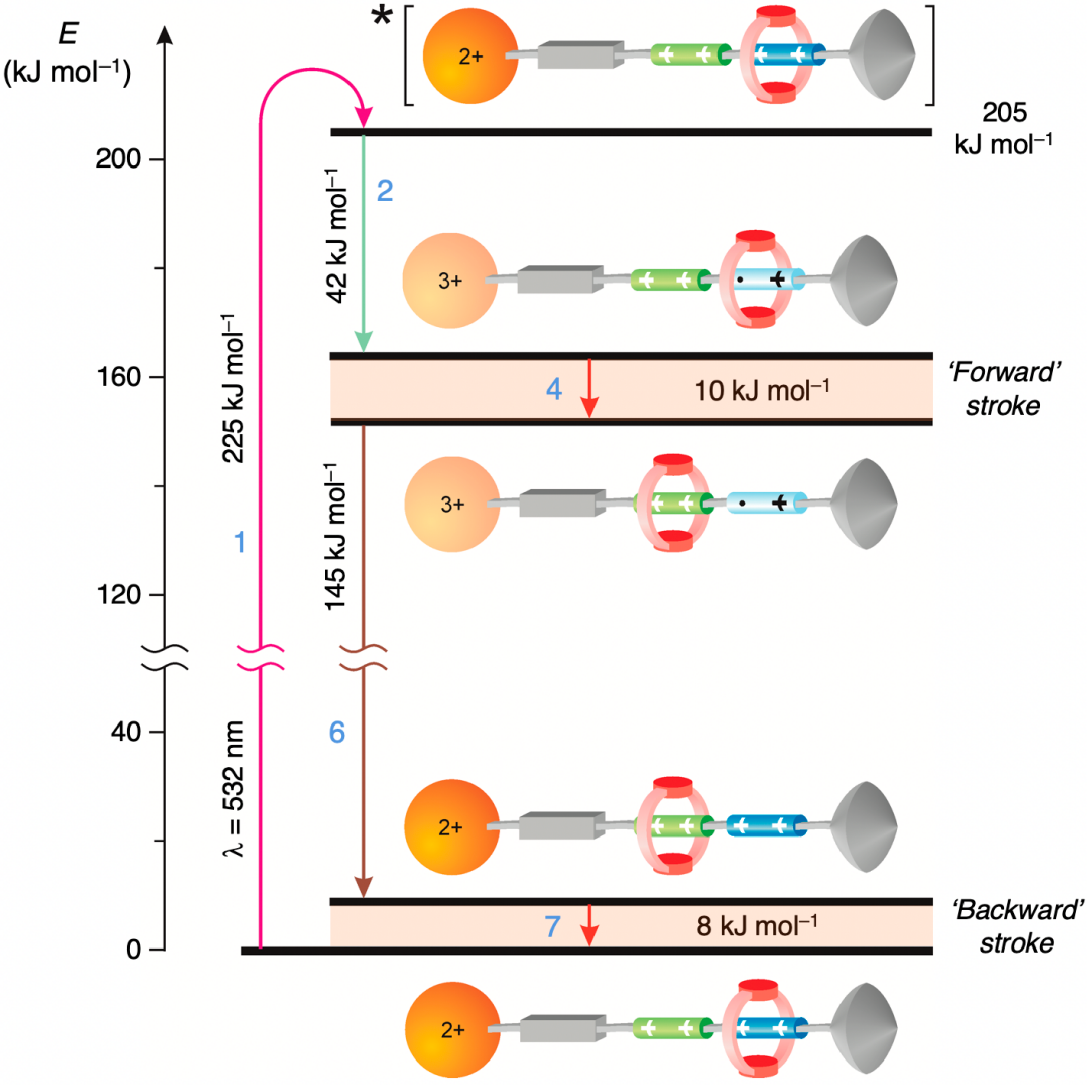
Schematic energy-level diagram for the operation of rotaxane **2** as a reversible autonomous molecular shuttle fueled by visible light (Figure 4). Arrows are color coded, and processes are numbered as in Figure 4. For more details see the text.

As it happens for macroscopic machines, the successful operation of this nanodevice relies on a proper synchronization of the processes that contribute to its mechanism (Figure 4), a result that requires a fine control of kinetics which can be achieved by careful structural design.^62^ Because of competition with undesirable energy-wasting processes (primarily, step 5), the overall quantum yield for ring shuttling (Φ_sh_) was found to be only 2% in acetonitrile at 303 K. The free energy available for the shuttling, which, in principle, could be used to do mechanical work, was estimated from thermodynamic data (redox potentials and equilibrium constants). As depicted in the energy-level diagram in Figure 5, the free energy change during ring shuttling is ca. −10 kJ mol^–1^ in the forward movement and −8 kJ mol^–1^ in the backward one. Indeed, most of the photon energy is dissipated to transfer electrons across the molecule. The fraction *F* of the excited state energy used to move the ring is about 9%, and the overall efficiency of light-to-mechanical energy conversion, η = Φ_sh_ × *F*, amounts to 0.2%. Considering that the full cycle is completed in 1 ms, the machine can potentially operate at a maximum frequency of 1 kHz and generate a mechanical power of 3 × 10^–17^ W per molecule. While such low figures appear disappointing, it should be noted that molecular shuttle **2** can utilize a free energy source (sunlight), and it is extremely stable and functions under mild environmental conditions (liquid solution at room temperature).

It should be noted, however, that while in both **1** and **2** a fraction of the incoming light energy input is indeed employed to move the ring, in the described experiments, the latter is not doing any mechanical work, as the energy is simply dissipated in the solution due to the viscous drag of the solvent. Another crucial issue, common to all molecular switches and shuttles, is that the reset of the ring occurs by traveling backward along the same coordinate of the forward motion. At the molecular level, this means that any effect performed on the environment during the forward movement (e.g., pushing against a force applied to the ring) would be undone by the backward step. Such a problem could be circumvented by coupling the shuttling motion with another process that desymmetrizes the operation cycle.^63^ A more insightful discussion on this aspect can be found in refs (27, 28, and 64).

Notably, compounds that can extend and contract their length under stimulation were developed by applying the paradigm of a controllable molecular shuttle to doubly interlocked rotaxane dimers ([c2]daisy chains).^65^ Systems of this kind were nicknamed “molecular muscles”^66,67^ because of the functional resemblance with the sarcomeres present in skeletal muscles.^8,9^ Rotaxane-based molecular muscles that can be reversibly interconverted between extended and contracted forms upon light irradiation were reported. These machines, which rely on the *E-Z* photoisomerization of azobenzene^68^ or stilbene^69^ units incorporated in the axles, were employed to make cross-linked polymers that exhibit macroscopic contraction–extension in response to light.^70^

### 3.2 Photoactivated Supramolecular Pumps

Molecular pumps can be defined as linear motors in which a molecular substrate is directionally transported with respect to the motor component.^71^ The transport must be active, that is, intrinsically performed by the motor using its energy source, without relying on external concentration differences; in principle, it should take place *against* a concentration gradient. In fact, molecular pumps are energy transducers capable of converting the energy input of the device into a chemical potential; in other words, they can use an external energy source to generate a nonequilibrium state.^72^ Conversely, passive transporters simply facilitate the system to relax to equilibrium by moving a cargo down a concentration gradient. The controlled transport of molecular and ionic substrates across biological membranes, that define and separate compartments, is a fundamental task for living organisms.^7−9^ The construction of artificial molecular species capable of performing such a function is motivated not only by the high basic science value but also by the potential for technological and medical applications.^73,74^ Such an endeavour can take significant advantage from the approaches pursued in the making of artificial molecular machines.^75−77^

**Figure 6:**
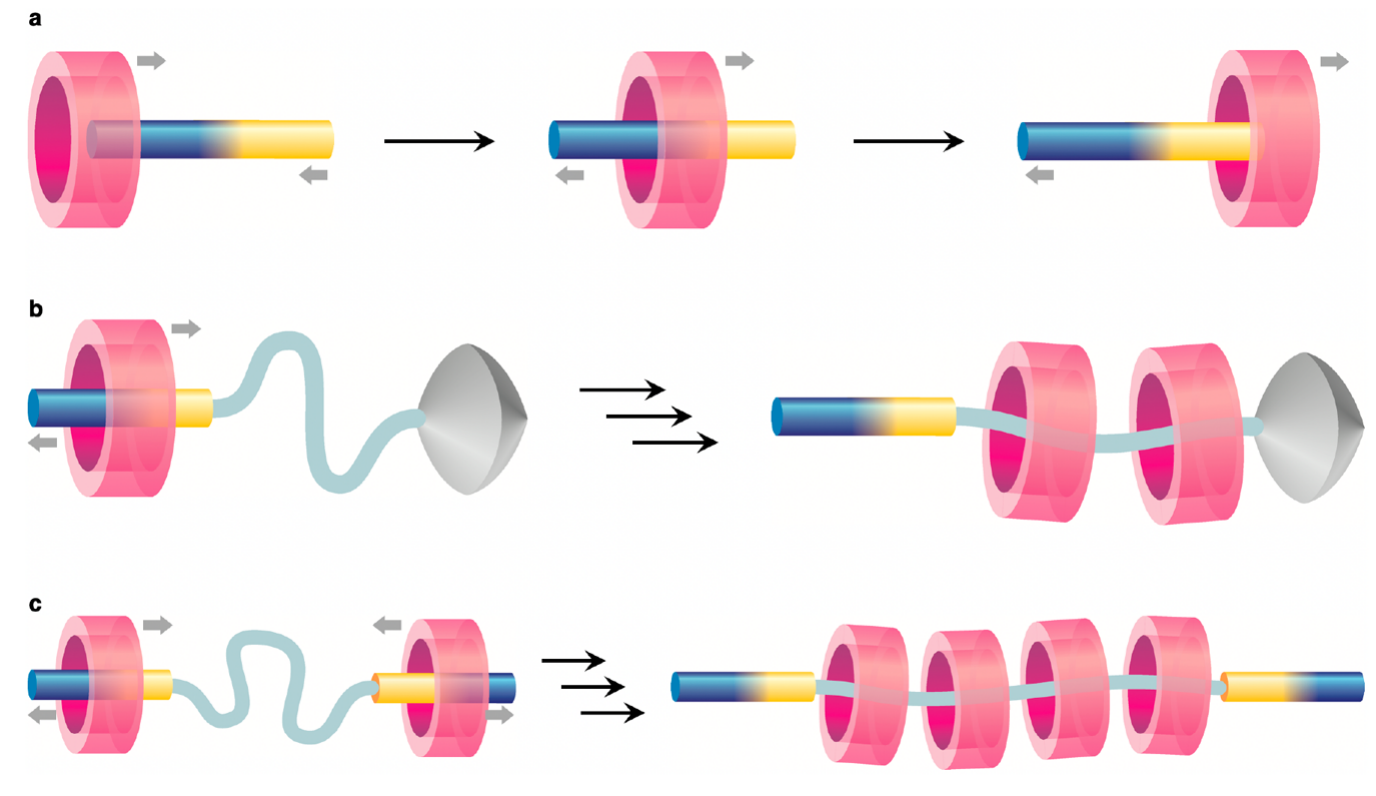
Molecular pump designs based on directionally controlled ring movements in (pseudo)rotaxane structures: unidirectional threading–dethreading in pseudorotaxanes (a) and ring accumulation in semirotaxane (b) and dual pump (c) architectures.

Artificial molecular pumps were obtained in recent years by following supramolecular strategies based on (pseudo)rotaxane architectures.^71,78^ By defining the axle as the “track” of the machine, the ring component may be directionally moved—that is, transported—along the track. It should be noted, however, that in a homogeneous solution none of the molecular components are connected to a fixed reference system, and only relative movements can be considered. Hence, the distinction between the “transporting” and “transported” components becomes purely conventional. Three classes of pumps were described so far;^79^ in the first one, the ring and axle components of a pseudorotaxane complex undergo unidirectional threading and dethreading. It can be envisioned that the ring becomes associated with the axle by passing over a specific extremity of the latter; successively, the ring dissociates by exiting from the opposite side (Figure 6a). Pumps of the second kind are semirotaxane architectures in which a macrocycle passes over the unstoppered end of the axle, travels directionally along the latter, and is eventually blocked in the stopper-terminated portion of the track (Figure 6b). A third design involves the construction of palindromic tracks that bear pump modules at both extremities, such that rings are pushed from both ends toward a central catchment region (Figure 6c).

In all cases, the pumping cycle can be repeated, resulting in the passage of successive rings along the axle in the pseudorotaxane species or in the accumulation of more than one ring on the axle in the semirotaxane or dual pump classes. In the former case, the threading–dethreading cycle can produce a net effect only if the transport involves spatially separated “departure” and “arrival” compartments. This result could be achieved, for example, by a membrane that provides the boundary of the compartments and the fixed support for the “transporting” component of the pump.^74−76^ Conversely, in the semirotaxane and dual pump cases, the macrocycle(s) can be pumped energetically uphill along the axle, which thus acts as a “reservoir” of ring(s) in a metastable state. Therefore, chemical energy can, in principle, be stored in the system, even in homogeneous solution. Artificial molecular pumps of the second and third kind are only available powered by chemical^80,81^ or electrochemical^82,83^ inputs.

Supramolecular pumps of the type shown in Figure 6a, powered by light, were realized by installing a photoreactive moiety in the molecular axle of the pseudorotaxane, such that its light-induced transformations affect both the thermodynamics and kinetics of the ring–axle interaction, giving rise to a ratchet mechanism that enables the rectification of the Brownian threading and dethreading movements.^78,84^

**Figure 7:**
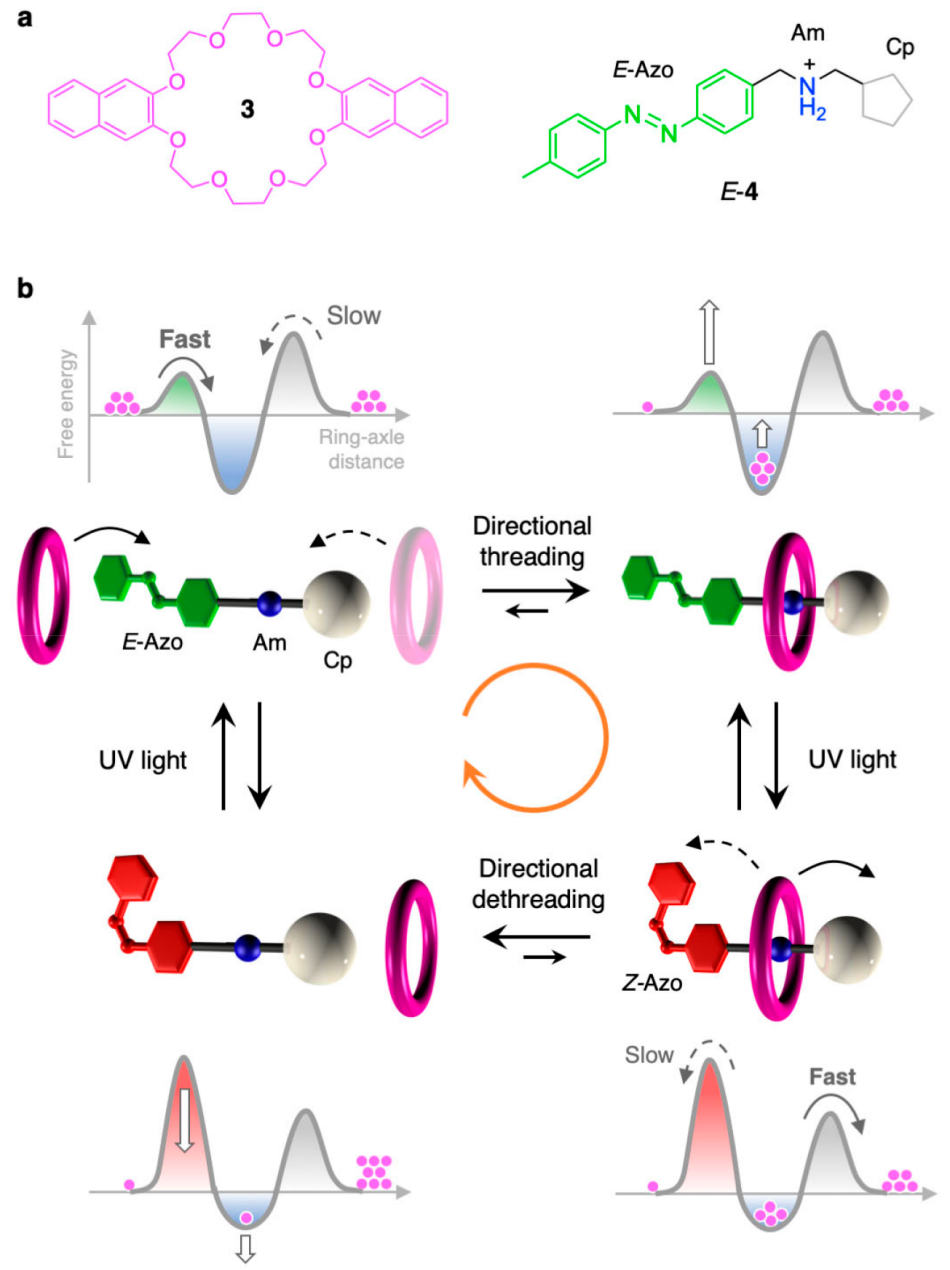
Structure formula of the molecular components (a) and schematic operation scheme (b) of an autonomous molecular pump fueled by light.^85^ The simplified free energy profiles corresponding to each structure in (b) are also shown.

The progenitor of this family of pumps^85^ consists of the aromatic crown ether **3** and the nonsymmetric molecular axle **4** (Figure 7a), which in turn comprises an azobenzene photoswitchable extremity (Azo), an ammonium recognition site (Am), and a photoinactive cyclopentyl end (Cp).^86^ In dichloromethane at room temperature, ring **3** and axle *E*-**4** form a very stable pseudorotaxane, on account of hydrogen bonding of the ammonium site of **4** with the oxygen atoms of **3**, and some π-stacking interactions involving the naphthalene and azobenzene aromatic moieties. Since the Azo unit in its *E* configuration exhibits a much smaller hindrance than Cp for the transit of the ring,^87,88^ a strong kinetic preference exists for threading through the Azo end, thus dictating the direction of the threading step (Figure 7b).

The isomerization of Azo to the *Z* form, triggered by the absorption of a UV or blue photon, has two key consequences: (i) the complex becomes less stable and(ii) the threading barrier at the Azo end is dramatically increased, making the Cp end the kinetically preferred extremity for the transit of the ring. As a result, the pseudorotaxanes undergo partial dethreading, and in doing so, the rings must escape from the Cp end (Figure 7b). Because both isomeric forms of azobenzene are photoreactive and absorb in the same spectral region, another photon, identical to the first one, can trigger the transformation of *Z*-**4** back to *E*-**4**, thereby closing the switching cycle.

The scheme in Figure 7b highlights the ability of the system to rectify Brownian motion by using the energy of photons and to repeat its working cycle autonomously under steady irradiation.^89^ Moreover, experiments showed that the pump components harness light energy to move progressively away from equilibrium. Indeed, the concentrations of the species measured at the photostationary state indicate that a net flux of species occurs along the closed reaction network in the clockwise direction (orange arrow in Figure 7b), which continues as long as photons impinge on the solution. In particular, the concentration of the *Z* complex is significantly increased with respect to its equilibrium value; as this species undergoes very slow dethreading, it is kinetically accumulated during cycling. Such a nonequilibrium state is referred to as dissipative because it requires a continuous input of energy to exist.^90^

The performance of the pump could be assessed by computer simulations of the cycle shown in Figure 7b, based on the experimentally determined rates of the thermal and photochemical reactions.^85^ Under the conditions employed, the cycling rate was 1.7 × 10^–10^ mol L^–1^ s^–1^ and the quantum yield was 2.3 × 10^–3^; the reciprocal of the latter number indicates that about 430 photons have to be absorbed to perform a full cycle. The ratio of the forward (clockwise) and backward (counterclockwise) rate constants shows that, on average, the pump functions in the “wrong” direction once every 160 cycles. This figure is related to the nonstandard free energy change of the system (Δ*G*) upon performing directional cycling, i.e., the energy stored in the reaction network under dissipative nonequilibrium conditions.^40^ Δ*G*, which also corresponds to the maximum amount of input energy that can be converted into useful work, was found to be −12.6 kJ mol^–1^ at 20 °C, that is, about 25% of the energy provided by ATP hydrolysis.^85^ Considering that 430 photons of 365 nm (326 kJ mol^–1^) are required to complete a cycle, the upper limit for the energy conversion efficiency is η = 12.6/(430 × 326) = 9 × 10^–5^. Such numbers show that the system is able to exploit only a tiny fraction of the input light energy because, not surprisingly, most of the energy is wasted into heat in excited-state vibrational relaxation processes.

More recently, the same behavior was observed with modified ring and axle components, provided that the key mechanistic elements depicted in Figure 7b are preserved.^91−94^ These results highlight the robustness and flexibility of this approach, whose appeal is further increased by its minimalist design, structural simplicity of the components, ease of preparation, stability, and reversibility.

### 3.3 Light-Powered Molecular Rotary Motors

**Figure 8:**
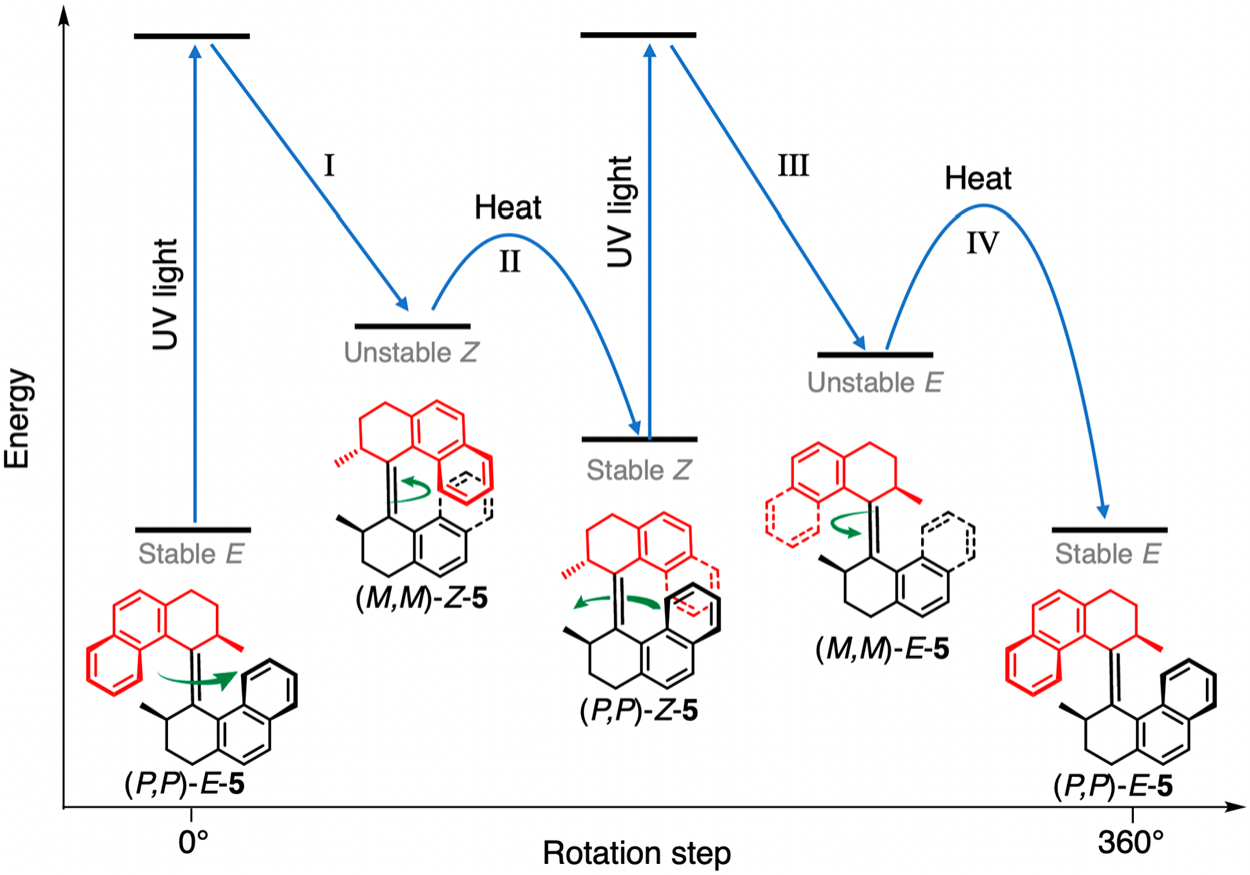
Molecular structures, mechanism, and simplified free energy profiles for unidirectional rotation in the overcrowded alkene (*R,R*)-**5**. Steps I and III are the *E-Z* isomerization processes, whereas steps II and IV are the thermal helix inversion processes. Adapted with permission from ref (29). Copyright 2017, Royal Society of Chemistry.

At the molecular level, the construction of rotary motors—i.e., machines that use energy to perform repeated unidirectional 360° rotations of one component with respect to the others—is particularly challenging because of the unidirectionality requirement. Artificial molecular rotary motors fueled by light were first obtained by exploiting the *E*-*Z* photoisomerization around a C=C double bond in overcrowded chiral alkenes.^95^ Let us consider the progenitor of the series (**5** in Figure 8) to describe the operation of this class of motors. Because of steric hindrance, the double bond system is constrained out of planarity, giving a helical shape to the molecule. Since the helicity can be either right handed (*P*) or left handed (*M*), a total of four stereoisomers exist for each enantiomer of **5** (e.g., the *R,R* one shown in Figure 8). The *E*-*Z* photoisomerization reactions are reversible and occur upon irradiation at appropriate wavelengths, whereas the thermal helix inversion processes (while maintaining the *E* or *Z* configuration) are irreversible, i.e., unidirectional. The tendency of the methyl substituents to adopt a less sterically demanding, energetically favorable axial orientation is the driving force for the directional rotation in the helix inversion. As shown in Figure 8, the 360° unidirectional rotation relies on a sequence of two energetically uphill light-driven isomerization processes and two energetically downhill thermal helix inversion steps. Indeed, the continuous irradiation at appropriate wavelengths (280–350 nm) of **5** at a sufficiently high temperature (60 °C) results in repeated rotations, and thus, the motor can operate autonomously under the supply of light energy.^94^

Overcrowded alkene rotary motors have evolved impressively over the past 20 years^96^ and were exploited in various ways to control functions and perform tasks.^97^ A key feature of these compounds is that the light-driven unidirectional rotation observed in a fluid solution is preserved when the motors are deposited on macroscopic or nanostructured surfaces, embedded in membranes, and incorporated in hard or soft materials.^32−36^ The robustness of the motor function is indeed one of the main reasons for the widespread use of these species in the search for applications of artificial molecular machines. Light-driven rotary motors based on different classes of molecules^98^—for example, imines,^99^ indigos,^100^ and catenanes^101^—have also been investigated.

The integration of molecular machines and motors with polymer chains has long been proposed as a promising strategy for harnessing the potential of the former to perform work.^32−34,64,66,67^ In recent years, the ability of covalent polymers embedding molecular machines to collect and amplify movement from the nanometer to the macroscopic scale was demonstrated.^66,67,70^

**Figure 9:**
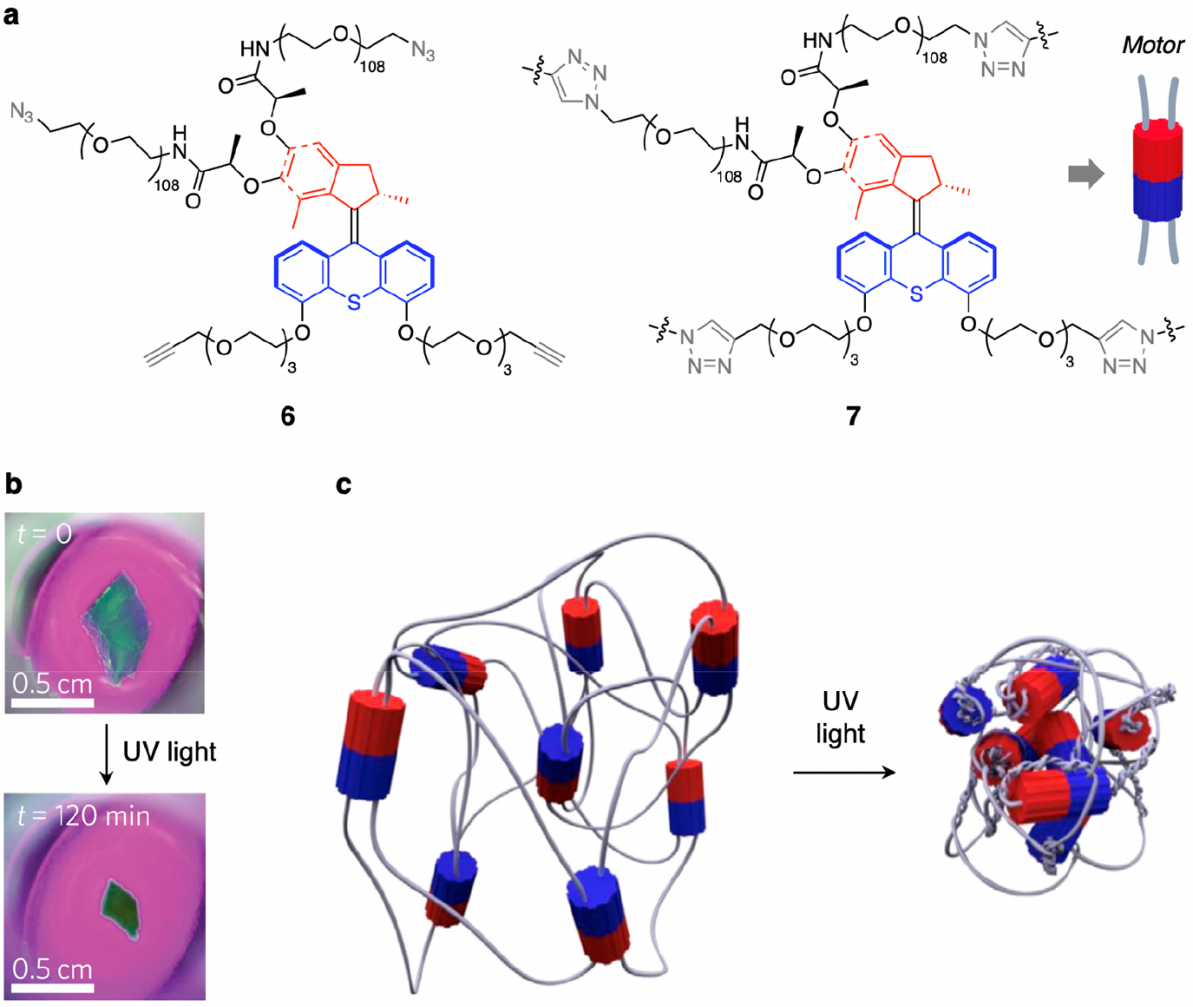
(a) Intermolecular “click” reaction between molecules of **6** affords the polymer-motor conjugate **7**. (b) Photographs showing the contraction of a piece of gel, consisting of **7** swollen with toluene, upon UV light irradiation. (c) Schematic representation of the braiding of the polymer chains of **7**, caused by the photoinduced rotation of the molecular motor units at the branching points, that results in the shrinkage of the gel. Adapted with permission from ref (101). Copyright 2015, Springer Nature.

Second-generation overcrowded alkene rotary motors were embedded in a polymeric gel, and the effect of UV irradiation on the resulting material was studied.^102^ Compound **6** (Figure 9a) was obtained by functionalizing the stator and rotor halves of the enantiopure motor with two alkyne-terminated oligomeric ethylene glycol chains and two azide-terminated poly(ethylene glycol) chains, respectively. The successive copper-catalyzed alkyne–azide cycloaddition (“click” reaction) performed on **6** under high concentration conditions afforded the cross-linked polymer **7** (Figure 9a) in which the ethylene glycol chains attached to different motors are connected together by means of triazole units. The polymer was found to form a gel in toluene at 10% w/w which was characterized by small-angle X-ray scattering (SAXS) and atomic force microscopy (AFM), supported by DFT calculations. The contraction of a millimeter-sized piece of the gel, caused by UV irradiation, could be clearly seen (Figure 9b). Such a behavior was explained by proposing that the entangled polymer chains are coiled because of the light-driven unidirectional rotation of the motor components (Figure 9c), an interpretation consistent with the results of control experiments performed on model compounds.^102^

It should be noted that this system exploits the unidirectional movement of a molecular motor driven autonomously by a single stimulus and is therefore substantially different from materials based on molecular mechanical switches, which are interconverted between two thermodynamic minima by different stimuli.^59,66,67,70^ As a matter of fact, the continuous photoinduced rotation of the motor units in **7** drives the system progressively away from thermal equilibrium; the energy of incident photons is thus converted into free energy of the entangled polymer chains, which lower their entropy. The free energy stored in the gel was estimated to be ca. 1 kJ mol^–1^ per motor turn, with an overall light-to-potential energy conversion efficiency of about 0.15%.^102^ This energy, however, could not be retrieved because the light-driven braiding of the polymer was irreversible.

**Figure 10:**
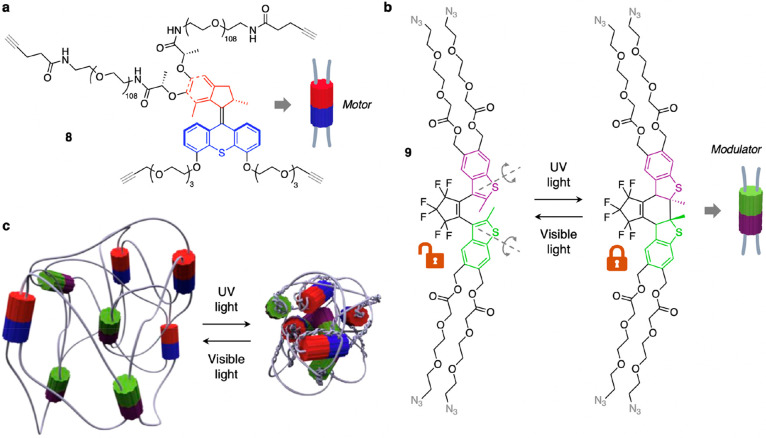
(a) Structure formula of the alkyne-terminated molecular rotary motor **8**. (b) Structure formula of the diarylethene-based compound **9** which, upon quadruple intermolecular “click” reaction with **8**, yields a cross-linked polymer similar to **7** (Figure 9a) that contains both a molecular motor and modulator units. The open (modulator unlocked; rotation axes shown in gray) and closed (modulator locked) forms can be interconverted by UV and visible light irradiation. (c) Schematic representation of the gel obtained by copolymerization of **8** and **9** and of the UV-induced braiding and visible light-induced unbraiding of its chains. Adapted with permission from ref (102). Copyright 2017, Springer Nature.

To address this issue, the design depicted in Figure 9 was improved by introducing diarylethene photoswitches in the gel as “modulator” elements^103^ that act as on-demand elastic releasers by unbraiding the polymer chains in the cross-linked network. For this purpose, compound **8** (similar to **6** but containing only alkyne ends) was copolymerized with the azide-terminated diarylethene derivative **9** (Figure 10a) to yield a polymeric gel analogous to **7** (Figure 9a) which contains both modulator and motor subunits. A key feature of this design is that the modulators can be activated at a wavelength different from that employed to operate the rotary motors. In particular, irradiation with UV light promotes the unidirectional rotation of the motors (Figure 9c), while the diarylethene units are in their closed (locked) form, thereby sustaining the coiling of the polymer chains caused by the motors. Conversely, visible light irradiation switches the modulators to their open (unlocked) form, while it is ineffective for the actuation of the motors (Figure 10). Free rotation around C–C single bonds in the diarylethene units can thus release the torsional energy accumulated in the braided polymer until thermodynamic equilibrium is reached. It is important to note that the reversibility of the process relies on the elastic properties of the polymer.

Upon irradiation with both UV and visible light, the motors coil the polymer chains, and the modulators allow their unwinding. By adjusting the relative intensities of UV and visible light, one can tune the winding and unwinding rates and overall determine whether the gel is contracting or expanding. When these rates are equal, a photostationary out-of-equilibrium state is reached. Hence, the energy stored in the material and its mechanical output can be tuned by optical modulation of the braiding and unbraiding frequencies. As discussed in the Introduction, unidirectional motion is necessary for a molecular machine to bring its surroundings away from equilibrium; collection and accumulation of the effects of such movements in a material, however, may lead to irreversible changes that prevent the exploitation of the harvested energy (see above). The strategy represented in Figure 10 provides a general solution to this problem by precise molecular design and paves the way toward the development of more complex molecular motor-based systems capable of converting light energy into potential and mechanical energy.

## 4 Conclusions and Perspectives

The study of artificial molecular machines and motors is an active and fascinating field of research that has now reached full maturity. This research has brought about significant innovation in chemistry, and it has increased its cross-disciplinary character by establishing new connections with physics, biology, materials science, and surface science. Nevertheless, the use of artificial molecular machines to accomplish specific tasks remains a challenging goal and provides a strong motivation for further research efforts. Since all molecular machines are fueled by an energy source, the management of energy—for example, its storage, retrieval, and conversion between different forms—is an obvious and most desirable function. Biomolecular machines beautifully accomplish this goal at both molecular (e.g., ATP synthase) and macroscopic (e.g., muscle myosin) scales.

Although artificial molecular machines cannot (yet) rival their biological counterparts in terms of complexity and efficacy, they are not limited to the blueprint established by nature. For example, the molecular machines described in this review are powered by light—a fact that is uncommon for biological devices but is highly advantageous from a technological viewpoint. Investigations focused on the use of artificial molecular machines for converting and storing energy are currently a rarity, and theoretical formulations describing nonequilibrium thermodynamics of photochemical reaction networks have just started to appear.^104^ The case studies discussed here show that a most critical point in this regard is the designed and effective integration of the molecular devices with their environment, in order for the input energy, part of which is used to produce directed motion at the molecular scale, to result in an effect on the surroundings which ultimately causes a sizable free energy change of the system or material.

Such a goal can be accomplished at different length scales. At the molecular level, energy conversion and storage with molecular machines requires a precise coupling of mechanical and chemical processes and the ability to generate nonequilibrium states that can persist over time as long as energy is supplied.^105,106^ At the micro- and macroscopic scales, strategies are required to organize molecular machines at interfaces, on surfaces, or in materials, such that the synchronous and cumulative activation of a large number of molecular devices allows the amplification of the effects of nanoscale motion up to larger scales.^107^ Clearly, a substantial research effort will be necessary to learn how to introduce molecular machines in complex structured matrices and get them to work, either individually or collectively, in a predictable and reliable way.

Another open problem is the exploitation of visible or near-infrared (IR) photons, which constitute the largest part of the Sun’s output power that reaches the surface of Earth, in the place of high energy, potentially harmful ultraviolet ones. A handful of artificial molecular machines powered with visible light are available (see, e.g., Figure 4), but their ability to process solar energy in the real world has not yet been demonstrated. Specifically, recently developed molecular rotary motors (e.g., Figure 8) operated with visible^100,108,109^ or near-infrared^110^ light would be extremely appealing to make active polymeric materials, such as those shown in Figures 9 and 10, capable of converting and storing the energy of sunlight. Last but not least, the photochemical and thermal stability of molecular machines in the long term is another issue of concern when real world applications are considered.

In conclusion, research in the past few decades has shown that the manifold characteristics of the interaction between light and molecules, together with the progress made in synthetic, supramolecular, and systems chemistry, can enable the realization of molecular machine-based systems with the potential for breakthroughs in energy conversion and storage. Indeed, the study of molecular assemblies that can harness solar energy in the form of UV, visible, or IR light is of fundamental importance to develop sustainable processes and materials arising from nanotechnology.
